# N-Formyl-L-aspartate mediates chemotaxis in sperm *via* the beta-2-adrenergic receptor

**DOI:** 10.3389/fcell.2022.959094

**Published:** 2022-09-23

**Authors:** Durva Panchal, Shweta Bhagwat, Priyanka Parte

**Affiliations:** ^1^ Department of Gamete Immunobiology, ICMR-National Institute for Research in Reproductive and Child Health, Mumbai, Maharashtra, India; ^2^ Department of Obstetrics and Gynecology, Washington University School of Medicine, St. Louis, MO, United States

**Keywords:** sperm chemotaxis, N-formyl-L-aspartate, beta-2-adrenergic receptor, capacitation, acrosome reaction, ICI-118, 551

## Abstract

Chemotaxis is a highly conserved physiological event required for directed sperm movement during fertilization. Recently, studies from our laboratory have identified N-formyl-L-aspartate (NFA) as a sperm chemoattractant. NFA is a known agonist for the beta-2-adrenergic receptor (β-2-AR) that regulates cAMP production and Ca^2+^ mobilization in somatic cells. As these downstream signaling molecules are also reported to be involved in sperm chemotaxis, in the present study we investigated the putative mechanism/s by which NFA may mediate chemotaxis. Toward this, the expression and localization of β-2-AR in sperm were studied by Western blot and indirect immunofluorescence, respectively. The responses of sperm to various concentration gradients of NFA and ICI-118,551, a β-2-AR specific antagonist, were evaluated using the microfluidics device-based chemotaxis assay. The intracellular concentration of Ca^2+^, on exposure to NFA, was analyzed using FURA-2 AM-based fluorimetric assay. Furthermore, the effect of NFA on sperm capacitation and acrosome reaction was evaluated using Western blot and immunofluorescence. NFA exhibited a bell-shaped dose-response curve typical of chemotaxis, with maximum response observed at 0.01M NFA, beyond which it was inhibitory; β-2-AR localization was seen on the sperm head and the mid-piece region of the flagella. Inhibition of sperm chemotaxis by ICI-118,551 confirms that sperm respond chemotactically to NFA *via* β-2-AR. Interestingly, at the concentration used for chemotaxis, NFA induced an increase in the intracellular Ca^2+^ but decreased cAMP in capacitating sperm. However, NFA *per se* did not induce capacitation as seen from the lack of effect on tyrosine phosphorylation and membrane potential of uncapacitated sperm. Acrosome evaluation of NFA-treated sperm using PSA-FITC staining showed no effect on the acrosome structure. Our data thus provide evidence indicating that NFA induces sperm chemotaxis and the chemotactic response of sperm to NFA from the ovulatory phase of oviductal fluid is mediated through the β-2-AR on sperm possibly via non-canonical signaling.

## Introduction

In mammals, during fertilization, only a small percentage of ejaculated sperm enter the oviduct and are actively guided by a combination of different guidance mechanisms before reaching the egg. One of the stages in gamete communication is sperm chemotaxis characterized by the release of chemical ligands from either the egg or cumulus cell or oviduct (Eisenbach, 1999). Several sperm chemoattractants such as RANTES (Isobe et al., 2002), atrial natriuretic peptide (Zhang et al., 2006; Bian et al., 2012), progesterone (Teves et al., 2009), allurin (Burnett et al., 2011), acetylcholine (Ko et al., 2012), and chemokine receptor CCR6 (Caballero-Campo et al., 2013) have been reported in various mammalian species. Sperm chemotaxis is maintained within a few millimeters in the vicinity of the egg and involves a complex series of molecular events that ensures the arrival of sperm toward an egg and fertilization (Guidobaldi et al., 2012).

For chemotaxis to occur in the vicinity of the egg, sperm must be functionally reprogrammed or capacitated in the female reproductive tract. This functional reprogramming enables the sperm to recognize the chemoattractants secreted either by the egg or its surrounding cells (Eisenbach, 1999). Sperm may have chemosensory receptors which likely get activated on encountering their cognate ligands. In mammalian species, olfactory receptors (Flegel et al., 2016) and taste receptors (Frolikova et al., 2020) have been of interest as molecular sensors for sperm-egg communication; however, their physiologically relevant ligands have not been identified yet. In general, receptor–ligand interaction in the ampulla of the oviduct leads to rapid downstream signal transduction resulting in sperm flagellar motor activation and regulation of motility to modulate its swimming path (Inaba, 2003). At molecular levels, sperm chemotaxis is associated with an increase in the intracellular concentrations of cAMP, cGMP (Shiba and Inaba, 2022), and Ca^2+^ (Oliveira et al., 1999; Teves et al., 2006). Despite identifying the chemical signals important for proper sperm function, our knowledge about the chemosensory receptors and their physiologically relevant ligands remains rudimentary.

Synthetically derived N-formylated peptides were first described to induce chemotaxis in human sperm (Gnessi et al., 1986a). There has been an increasing interest in these synthetically derived analogs due to their inherent potential to induce chemotaxis in neutrophils (Southgate et al., 2008a) and different bacterial species such as *E.coli* (Bi et al., 2013a) and *P.aeruginosa* (Bufe et al., 2015a). Recent studies from our group identified NFA as a chemoattractant using rat sperm. NFA levels were significantly higher in the ovulatory phase of oviductal fluid than in the pre-ovulatory phase (Bhagwat et al., 2021a). This is an integrative study deciphering the possible mechanism of NFA-mediated sperm chemotaxis. Furthermore, we also explored whether NFA could influence other crucial events in sperm such as capacitation and acrosome reaction. The findings of this study will aid in understanding the role of NFA and other putative chemoattractants in enhancing the fertilizing ability of sperm.

## Materials and methods

### Chemicals and reagents

Dulbecco’s Modified Eagle Medium (DMEM) containing 4.5 g/L D-glucose, L-glutamine, 25 mM HEPES without phenol red, dimethylsulphoxide, Fura-2 AM, pluronic F-28, Triton X-100, CaCl_2,_ MnCl_2,_ and antagonist ICI-118,551, and 2-D quant kit were obtained from Sigma- Aldrich, USA. Chemicals used in the preparation of 2-D cell lysis buffer and sodium dodecyl sulfate-polyacrylamide were from HiMedia, India. NFA was procured from PureSynth Research Chemicals, Canada. The Femto-Plus Chemiluminescence detection kit and pre-stained protein markers were acquired from Thermo Fisher Scientific, USA. EDTA-free protease and broad-spectrum phosphatase inhibitor cocktail including Tyr, Ser/Thr phosphatases were obtained from Roche, Germany. The antibodies used in this study were as follows: rabbit anti-β-2-AR antibody purchased from BoosterBio, USA; mouse anti-α-tubulin antibody, mouse phosphotyrosine antibody, and PSA-FITC obtained from Sigma Aldrich, USA; HRP- and FITC-conjugated swine anti-rabbit and goat anti-mouse antibodies were from Dako-Agilent, USA.

### Ethics approval

Adult male Wistar rats, 3–4 months of age were used in this study. Three rats were housed per cage and maintained in an atmosphere of 14 h light and 10 h dark in the animal breeding facility at the National Institute for Research in Reproductive and Child Health (ICMR-NIRRCH). Food and water were provided *ad libitum.* All the animal care practices and experimental procedures were approved by the Institutional Animal Ethics Committee (IAEC) of ICMR-NIRRCH.

### Sperm preparation

The cauda epididymis of 3-months old sexually mature male rats were excised in sterile tubes containing DMEM, and sperm were allowed to swim up by incubation at 37°C for 15 min in an atmosphere of 5% CO_2_ and 95% air. The supernatant containing actively motile sperm was carefully separated and evaluated for concentration and motility. Sperm concentrations were greater than 50 × 10^6^ cells/mL and motility was over 70%. 2 × 10^6^ cells were then incubated in DMEM alone or for capacitation in DMEM supplemented with 2.5% bovine serum albumin (BSA) for 2.5 h at 37°C in an atmosphere containing 5% CO_2_ and 95% air (Bhagwat et al., 2018). Motility was assessed at 0 and 2.5 h of capacitation incubation and at the end of the chemotaxis experiment to ensure that the motility was more than 60% throughout the experiment. The effect of antagonist ICI-118,551 on chemotaxis was evaluated after exposing capacitating sperm to the antagonist for 30 min before the chemotaxis assay.

### Sperm motility assay

Sperm motility was analyzed using a motility chamber prepared as reported previously (Bhagwat et al., 2018). Briefly, the motility chamber was prepared by wrapping four layers of scotch tape on a glass slide (3 cm apart, height ∼200 microns). A suspension of 1:50 diluted sperm was loaded into a motility chamber pre-warmed at 37^°^C and a coverslip (22 × 40 mm) was placed on the slide. The slide was mounted on an optical microscope equipped with an sCMOS camera. Sperm motility was analyzed by capturing videos at ×10 magnification (8-bit MPTIFF, 2058 × 2018 pixels at 45 fps for 10 s). For each experimental set, ∼10 movies each of 10 s durations were recorded and at least 200 sperm were analyzed. Sperm viability was checked by staining sperm with 0.5% Eosin Y. Those that stained pink were counted as non-viable.

### Sperm chemotaxis assay

Sperm chemotaxis was analyzed using the microfluidic device developed in-house and as reported previously (Bhagwat et al., 2018). Briefly, for chemotaxis assay, the device was set in a chamber maintained at 37^°^C mounted on the stage of a laser scanning confocal microscope (LSCM; LSM780, Carl Zeiss Microscope, Thornwood, NY, USA). Different concentrations of NFA (0.001, 0.005, 0.01, or 0.02 M) were allowed to form gradients in DMEM in the test channels of the device by maintaining a constant flow rate of 1 μL/min using a syringe pump (NE-1000, New Era Pump System Inc., Farmingdale, NY, USA). Similar incubations in media alone served as the control in every experiment. Next, 2 × 10^6^ capacitating sperm were added to the cell reservoir. After stabilization of the flow and gradient for 10 min, sperm motion in the test channels was captured at ×10 magnification using an sCMOS camera (8-bit MPTIFF, 2058 × 512pixel) at 90 fps for 15 sec. During each experimental set, ∼30 movies each of 15s duration were recorded and at least 200 sperm were analyzed.

### Estimation of chemotaxis

Chemotaxis was determined by assessing the changes in sperm motion in response to gradients formed by NFA in the test channel. The total number of sperm moving toward and against the gradient concentrations of 0.001, 0.005, 0.01, or 0.02 M NFA and DMEM, was estimated. The percentage of sperm swimming in the ascending direction of the gradient was calculated for each gradient concentration of NFA and compared to its respective DMEM control. The speed bias of sperm swimming toward a chemoattractant gradient was assessed by calculating the straight-line velocity (VSL). Individual sperm traveling in the test channel were tracked for their VSL using the manual tracking plugin in Image-J software (v1.50b; NIH, USA). The net distance (d) was calculated using the formula $d=\sqrt{{\left(x_{2}-x_{1}\right)}^{2}+{\left(y_{2}-y_{1}\right)}^{2}}$ where $\left(x_{1\;},y_{1\;}\right)$ and $\left(x_{2\;},y_{2\;}\right)$ are the x and y coordinates of initial and final position, respectively, obtained while tracking individual sperm and was normalized to time (Schneider et al., 2012).

### Protein extraction and western blotting

Caudal sperm were obtained as described earlier in this section. Sperm pellets were washed thrice with 0.1 M phosphate buffer saline (PBS), pH 7.2, by centrifugation at 800 *g* for 30 min at 4°C. Sperm lysates were prepared by re-suspending the pellets in 2D-lysis buffer containing 7 M urea, 2 M thiourea, 4% CHAPS, and 30 mM Tris, supplemented with protease and phosphatase inhibitors, for 18 h at 4°C, followed by homogenization using a Fast-prep-24 Homogenizer (MP, Biomedicals, Irvine, United States). The homogenized lysates were incubated for 30 min at 4°C followed by centrifugation at 1,000 *g* for 30 min at 4°C to collect the supernatant. The total protein concentration in the supernatant was estimated using the 2-D quant protein estimation kit. A total of 40 µg of the protein lysate was electrophoresed on 10% SDS-PAGE and transblotted to a nitrocellulose membrane (Pall Biosciences, United States). Nonspecific binding was blocked by incubating the membranes with 5% non-fat dried milk (NFDM) in Tris-buffered saline containing 0.1% Tween (TBST) for β-2-AR, or with 5% BSA in 0.1% TBST for phospho-tyrosine, at room temperature (RT) for 1 h. The membranes were incubated overnight at 4°C with either anti-β-2-AR antibody diluted 1:500 in TBST containing 1% NFDM (TBST-NFDM) or with anti-phosphotyrosine antibody diluted 1:1,000 in TBST containing 1% BSA (TBST-BSA). The membranes were washed three times for 5 min each in 0.1% TBST and incubated for 1 h at RT with either 1:3000 HRP-conjugated swine anti-rabbit or 1:5000 HRP-conjugated goat anti-mouse secondary antibodies diluted in TBST-NFDM or TBST-BSA, respectively. The membranes were then washed five times for 5 min each and the protein bands were detected using a chemiluminescence-based detection system. To account for protein load, the same blots were probed with 1:10,000 diluted goat anti-α-tubulin antibody diluted in TBST-NFDM after stripping off the bound specific antibodies by two washes for 10 min each with stripping buffer, pH 2.2, containing 0.2 M glycine, 3.5 mM SDS, 8 mM Tween-20, and 0.3 mM HCl. The membranes were then washed twice for 10 min each with 0.1 M TBS, followed by two washes of 5 min each with 0.1% TBST and finally, two washes with 0.1 M TBS for 10 min each. The membranes were processed further as described previously. The lane intensity of tyrosine-phosphorylated proteins was measured and normalized to the band intensity of the loading control α-tubulin and the data were quantified using Image-J software (NIH, US).

### Indirect immunofluorescence

For IIF, sperm were resuspended in 0.1 M PBS, such that their final concentration was 2 × 10^6^ cells/mL. A total of 10 μL of the sperm suspension was spread uniformly on 0.1% poly-L-lysine-coated glass slides, air-dried and fixed in 95% (w/v) acetone for 3 min followed by 95% (w/v) methanol for 1 min at 4°C. Cells were either used intact or permeabilized with 0.1% (w/v) Triton-X-100 and 1% (w/v) glacial acetic acid. Non-specific binding was eliminated by incubating the slides with 3% BSA for 1 h at RT. The slides were then incubated overnight at 4°C with rabbit anti-β-2-AR, diluted 1:50 with 0.1 M PBS. The slides were washed three times for 10 min each with 0.1 M PBS at RT under slow rocking conditions. The slides were then incubated with 1:200 diluted FITC-conjugated swine anti-rabbit antibody and 0.01% DAPI (Sigma Aldrich, United States) for 1 h at RT in dark. Finally, the slides were washed thrice for 10 min each with 0.1 M TBS and mounted using Prolong-Gold (Sigma Aldrich, US). The slides were examined using an epifluorescence microscope (LSCM; LSM780, Carl Zeiss Microscope, Thornwood, NY, United States).

### PSA-FITC staining

The effect of NFA on the acrosome of capacitating sperm was assessed by the PSA-FITC staining method. 2 × 10^6^ sperm were allowed to capacitate by incubating them in DMEM supplemented with 2.5% BSA, for 2.5 h at 37°C in an atmosphere of 5% CO_2_ and 95% air. After incubation, the sperm were treated with either 0.01 M NFA or 10 μM progesterone for 30 min at 37°C in an atmosphere of 5% CO_2_ or 95% air. Capacitated sperm incubated with DMEM for 30 min served as controls. Next, 10 μL of each sample was uniformly spread on 0.1% poly-l-lysine-coated glass slides, air-dried, fixed with 95% (w/v) acetone for 3 min followed by 95% (w/v) methanol for 1 min, and incubated with 3% PSA-FITC (3 μg/100 μL) in dark for 30 min at RT. The slides were then washed thrice for 10 min each with 0.1 M PBS and mounted using Prolong-Gold. The slides were examined using an epifluorescence microscope. For each sample, 150–200 sperm were analyzed and classified as acrosome intact (AI) for sperm showing intense fluorescence over the sperm head indicative of an intact acrosome, or as Acrosome reacted (AR) for those with low or absent fluorescence over the head, typically seen in an acrosome-reacted sperm.

### Intracellular Ca^2+^ measurement in sperm

The intracellular Ca^2+^ [Ca^2+^]_I_ levels in sperm were measured using Fura-2AM, following an established protocol (Bhagwat et al., 2021a). Briefly, 10^7^ capacitating sperm were incubated in DMEM with or without the β-2-AR antagonist ICI-118,551 for 30 min at 37°C. They were then loaded with 5 μM Fura-2AM and incubated at 37^°^C for 45 min in an atmosphere of 5% CO_2_ and 95% air with intermediate mixing. After incubation, the dye-loaded cells were washed thrice with DMEM by centrifugation at 600 *g* for 30 min at 37^°^C to remove unbound Fura-2AM. 2 × 10^6^ dye-loaded sperm were then resuspended in Ca^2+^ containing Tyrode’s buffer with 0.001, 0.005, and 0.01 M NFA in a 96-well black polystyrene microtiter plate (Labsystems, India) at a final volume of 200 μL. Sperm were evenly resuspended by mixing for 30 s in the spectrofluorimeter. Fura-2AM excitation was set at 380 and 340 nm to determine the free and bound calcium, respectively. Fluorescence readings were recorded at intervals of 15 s for 15 min and cells were lysed with 1% Triton-X-100 followed by the addition of 3 mM CaCl_2_ for F_max_ and 2 mM MnCl_2_ for F_min_. The [Ca^2+^]_I_ concentration was calculated using the formula [Ca^2+^]_I_ $=\frac{Kd\;\left(F-Fmin\right)}{\left(Fmax-F\right)}$ where K_d_ is the dissociation constant, F is the fluorescence reading calculated as the ratio of 340/380, and F_max_ and F_min_ are the maximum and minimum calcium levels, respectively, over the incubation period of 15 min.

### Intracellular cAMP measurement in sperm

The intracellular levels of cAMP in sperm were measured using direct competitive ELISA (cAMP assay kit, ADI-900-066A, Enzo life science, US). Next, 10^6^ cells were incubated with either DMEM, DMEM supplemented with 2.5% BSA, or 0.01 M NFA at 37^°^C for 2.5 h in an atmosphere of 5% CO_2_ and 95% air. After incubation, 100 μL of each cell sample was added to 200 μL of 0.1 N HCl and homogenized using a Fast-prep-24 homogenizer. The cell lysate was centrifuged at 600 g for 5 min at 37^°^C. The resultant supernatant was then used for competitive ELISA as per the kit protocol.

### Sperm membrane potential measurement

The fluorimetric measurement of sperm membrane potential was carried out using a voltage-sensitive dye, DiSC_3_, using a published protocol (Baro Graf et al., 2020). Briefly, 10^7^ sperm were incubated for 2.5 h in either DMEM supplemented with 1% BSA, DMEM supplemented with 2.5% BSA, or with 0.01 M NFA and 1% BSA. From each group, 2 × 10^6^ sperm/200 μL were transferred to a black 96-well polystyrene microtiter plate and loaded with 1 μM DiSC_3_ solubilized in 1% DMSO. The plate was transferred to a spectrofluorimeter with gentle mixing for 10 s and the fluorescence was measured at 37°C at excitation/emission wavelengths of 620/670 nm, respectively. Internal calibration for each determination compensates for the variable that can influence the absolute fluorescence values. Calibration of the DiSC_3_ assay was carried out using 1 μM valinomycin dissolved in 1% DMSO and sequential addition of 1.67, 3.40, 5.95, and 10.20 μL of 2 M KCl. The theoretical Em values were calculated using the Nernst equation considering 120 mM as the internal K^+^ concentration. The final membrane potential was calculated by linearly interpolating the theoretical Em values against the arbitrary fluorescence units at each time point.

### Statistical analysis

Statistical analysis was done using GraphPad Prism 9.0.4 software (GraphPad Software Inc.). Results are expressed as mean ± SD or SEM. One-way ANOVA was used to determine the difference between the groups with Dunn’s post hoc test for multiple comparisons. When the experiments had more than one variable, two-way ANOVA was used for statistical analysis. The difference was considered to be statistically significant when the *p*-value was less than or equal to 0.05.

## Results

### NFA demonstrates a bell-shaped dose-dependent response typical of a chemoattractant

**FIGURE 1:**
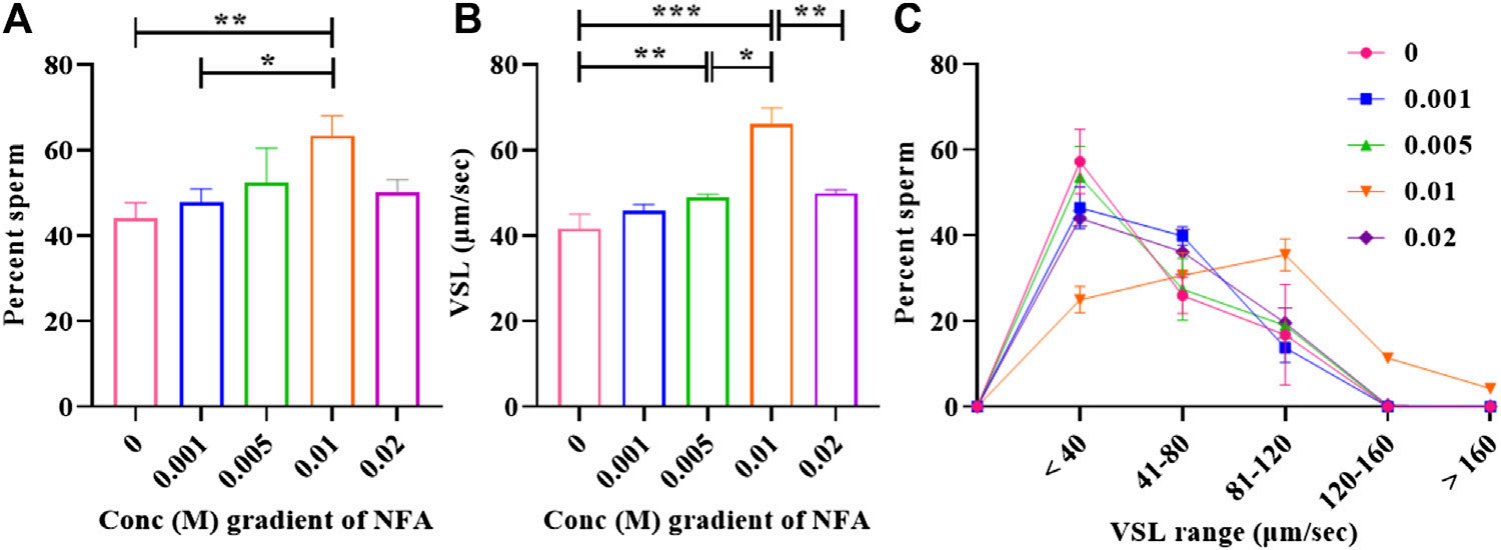
Bell-shaped dose-response of sperm to NFA. Chemotaxis was studied using the microfluidics device at gradient concentrations of 0, 0.001, 0.005, 0.01, and 0.02 M NFA after capacitating sperm for 2.5 h. Sperm directionality is represented as the percentage of sperm entering the transverse channel and moving toward the ascending concentration gradient of NFA **(A)**, sperm VSL **(B)**, and frequency distribution of sperm VSL in response to increasing gradients of NFA **(C)**. Each column represents mean ± SEM for the respective gradient tested (***p < 0.005; **p < 0.01; *p < 0.05). The results presented are cumulative from three independent experiments.

Recent studies by our group reported sperm chemotaxis at a concentration gradient of 0.01 M NFA (Bhagwat et al., 2021a). In the present study, we evaluated the response of sperm to concentration gradients lower and higher than 0.01 M NFA using microfluidic device-based chemotaxis assays. To examine whether and how higher or lower concentration gradients of NFA influenced the sperm numbers entering the transverse channel, we calculated the percentage of sperm moving toward and against gradients formed by 0, 0.001, 0.005, 0.01, and 0.02 M NFA. The percentage of sperm responding positively to NFA increased as the gradients got steeper up to a concentration of 0.01 M, beyond which, i.e., at 0.02 M NFA, there was a decrease observed (Figure 1A). The results demonstrated a bell-shaped dose-dependent response curve (typical of chemotactic cell behavior), with significant response seen at a concentration gradient of 0.01 M NFA in comparison to the DMEM control (**p < 0.01) and in comparison, to a gradient of 0.001 M NFA (**p < 0.05).

Increase was also observed in the mean straight-line velocity (VSL) of sperm moving toward increasing concentration gradients of 0.001, 0.005, and 0.01 M, with statistical significance observed at 0.005 M (**p < 0.01) and 0.01 M (***p < 0.005) as compared to the DMEM control. A significant decrease in the mean VSL was seen at a gradient of 0.02 M NFA (**p < 0.01) as compared to that seen with 0.01 M NFA. The VSL at the 0.02 M gradient of NFA was comparable to that seen in the DMEM control, 0.001 M, and 0.005 M NFA (Figure 1B). A frequency distribution plot for sperm velocities at different concentrations of NFA showed that the sperm population exposed to a gradient formed by 0.01 M NFA had a higher percentage of sperm moving at the VSL range 81–160 and >160 μm/sec and a lower percentage of sperm in the VSL range of <40 and 41–80 μm/sec as compared to other concentrations (Figure 1C). Data for each experimental set are provided in Supplementary Table S1.

### β-2-AR is present on the sperm surface

**FIGURE 2:**
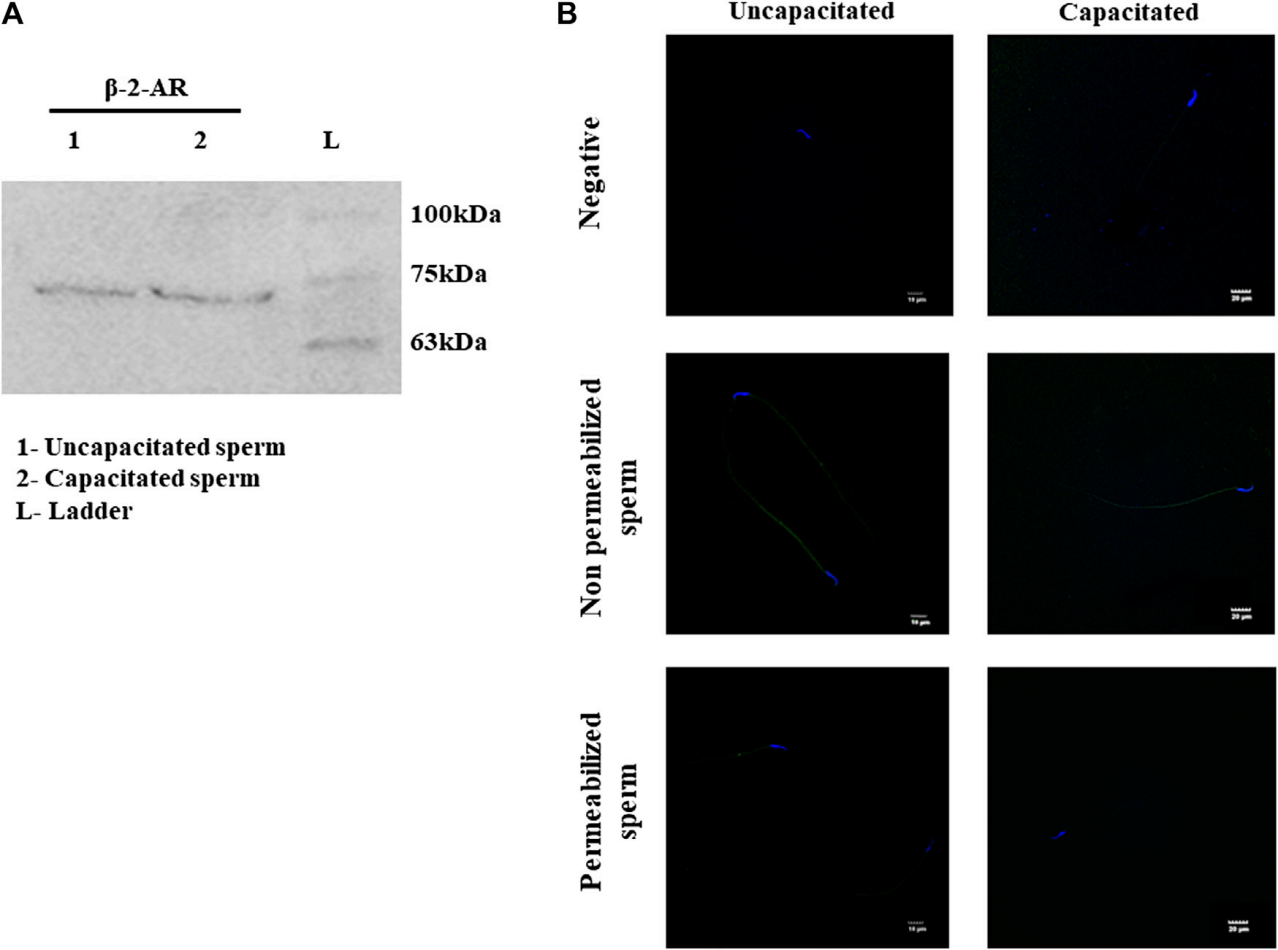
Expression and localization of β-2-AR in sperm. Lysates of uncapacitated and capacitated sperm were electrophoresed on 10% SDS PAGE and probed for β-2-AR by Western blot analysis, using antibodies specific for β-2-AR **(A)**. The complete blots and their ponceau profiles are provided in Supplementary Figure S1. IIF localization shows the presence of β-2-AR (green) in uncapacitated and capacitated intact and permeabilized sperm **(B)**. Nuclei of sperm were stained with DAPI (blue). The negative control shows sperm incubated with only the secondary antibody. This experiment was done in triplicates using sperm from three individual rats.

NFA is patented as a β-2-AR agonist (US-2005261338-A1, US-2007179179-A1, and US-2008269344-A1). Additionally, N-formylated peptides have been suggested to demonstrate chemotaxis toward human sperm (Gnessi et al., 1986a), neutrophils (Naccache et al., 2000a; Southgate et al., 2008a), and bacterial species such as *Pseudomonas aeruginosa* (Ijiri et al., 1994a; Bufe et al., 2015a) and *E. coli* (Bi et al., 2013a). Studies from our group have reported significantly increased concentrations of NFA in the ovulatory phase compared to the pre-ovulatory phase-rat oviductal fluid. Therefore, we first investigated the presence of β-2-AR in rat sperm. By Western blot analysis, a specific band at ∼72 kDa was detected for β-2-AR in uncapacitated and capacitating sperm lysates (Figure 2A). Similarly, evaluation of human sperm lysates detected proteins of molecular weights ∼95 and ∼55 kDa, in addition to the ∼72 kDa band (Supplementary Figure S1). Furthermore, we studied the localization of β-2-AR in uncapacitated and capacitated sperm using IIF. A positive signal for β-2-AR was seen on the head and the mid-piece region of sperm, with decreasing or fainter fluorescence on the principal piece and end piece. On permeabilization of sperm with 0.1% Triton X-100 and 1% glacial acetic acid, the intensity of the fluorescence signal diminished indicating that β-2-AR is present on the sperm surface. Although the immunofluorescence data are not quantitative, there was no discernible difference in β-2-AR intensities between capacitated and uncapacitated sperm (Figure 2B).

### β-2-AR antagonist blocks NFA-mediated sperm chemotaxis

Having obtained evidence for the presence and location of β-2-AR on sperm, we next looked for evidence on whether or not NFA mediates its chemotactic effect *via* β-2-AR, using the β-2-AR-specific antagonist ICI-118,551. Toward this, the effective concentration of ICI-118,551 that was not lethal to sperm *per se* was determined by quantifying sperm motility and viability immediately and after 15, 30, and 45 min post-incubation with 0.01, 0.1, and 1 µM ICI-118,551. The significance of differences between treatment groups was analyzed by 2-way ANOVA. Sperm motility was significantly increased after exposure to 0.01 M NFA compared to the DMEM control at all the time points tested. A slight yet significant reduction was seen with 0.01 µM ICI-118,551 compared to that with NFA immediately on incubation but not at later time points; however, it was comparable to the media control. At higher concentrations of ICI-118,551, it significantly and progressively reduced from 15 min onward with respect to that seen with DMEM and NFA (Table 1). On the other hand, sperm viability was compromised after 30 and 45 min incubation only with 1 µM ICI-118551 (Table 2). On the basis of these data, 0.01 µM ICI-118551 was identified as a suitable concentration and used for subsequent experiments to test whether it inhibits NFA-mediated chemotaxis.

**TABLE 1:** Percent motility of sperm on exposure to different concentrations of ICI-118,551.

Time (min)	DMEM	0.01 M NFA	0.01 µM ICI-118,551	0.1 µM ICI-118,551	1 µM ICI-118,551
0	58.90 ± 0.46	61.61 ± 0.51^a^ ^*^	58.75 ± 0.20^b^ ^*^	57.73 ± 0.96	57.42 ± 1.29
15	51.60 ± 1.40	59.36 ± 1.12^a^ ^*^	54.12 ± 1.32	47.50 ± 2.04^a^ ^*^	44.96 ± 0.57^a^ ^*,^ ^b^ ^**^
30	47.82 ± 0.95	57.74 ± 0.61^a^ ^**^	51.75 ± 2.24	45.32 ± 0.95^a^ ^*^	41.71 ± 0.88^a^ ^**,^ ^b^ ^***^
45	41.26 ± 0.57	52.89 ± 1.34^a^ ^*^	45.44 ± 2.39	38.50 ± 0.54^a^ ^*^	33.02 ± 1.20^a^ ^*,^ ^b^ ^***^

a significant compared to DMEM.

b significant compared to 0.01 M NFA.

(*p < 0.05; **p < 0.005; ***p < 0.001).

**TABLE 2:** Percent viability of sperm on exposure to different concentrations of ICI-118,551.

Time (min)	DMEM	0.01 M NFA	0.01 µM ICI-118,551	0.1 µM ICI-118,551	1 µM ICI-118,551
0	66.33 ± 3.85	66.00 ± 1.63	65.66 ± 3.39	65.33 ± 3.30	64.33 ± 4.19
15	64.00 ± 2.44	63.33 ± 2.62	66.33 ± 4.11	64.66 ± 2.62	55.00 ± 2.82
30	63.66 ± 2.62	62.66 ± 0.94	61.66 ± 2.62	65.00 ± 2.94	53.00 ± 5.71 ^a^ ^*^
45	57.33 ± 0.47	64.00 ± 2.44	60.00 ± 0.81	56.66 ± 1.70	44.67 ± 3.39 ^a^ ^*^

a significant compared to 0.01 M NFA.

(*p < 0.05).

**FIGURE 3:**
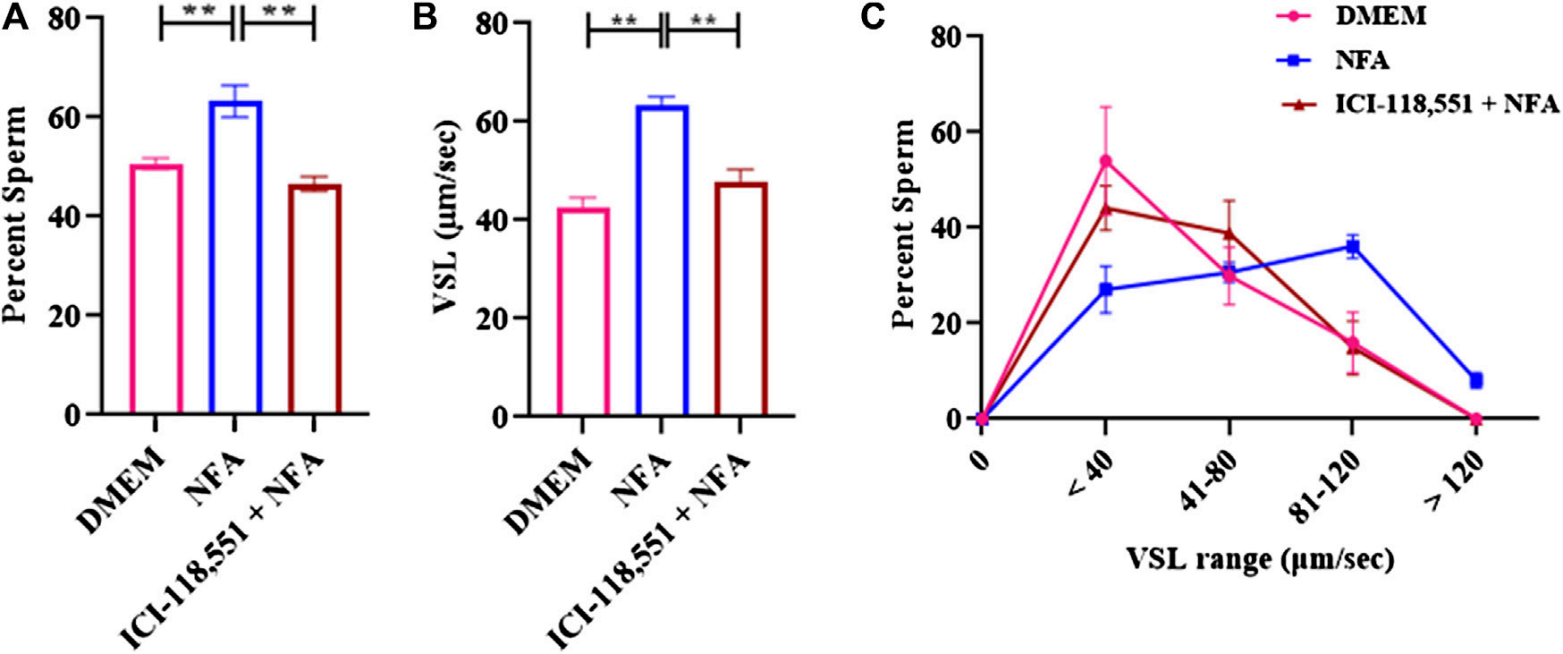
β-2-AR antagonist blocks sperm chemotactic responses to NFA. The effect of β-2-AR antagonist on sperm chemotaxis was studied after 30 min exposure of capacitating sperm to 0.01 μM ICI-118,551 before exposing them to a gradient of 0.01 M NFA and evaluating chemotaxis in the microfluidics device. Sperm directionality is presented as the percentage of sperm moving toward the ascending concentration gradient of NFA in the transverse channel **(A)**; sperm VSL **(B)**; and frequency distribution of sperm VSL under these conditions **(C)**. Each column represents mean ± SD (**p < 0.01). The results represented are cumulative from three independent experiments.

Chemotaxis to NFA in the presence of 0.01 µM ICI-118,551 was assessed by incubating sperm for 2.5 h with BSA to capacitate them followed by incubation with or without 0.01 µM ICI-118,551 for 30 min and then determining their response to a gradient of 0.01 M NFA in the chemotaxis assay. The percentage of sperm entering the transverse channel toward the ascending gradient of 0.01 M NFA and their VSL was determined. After treatment with 0.01 µM ICI-118,551, the percentage of sperm responding to an ascending gradient of 0.01 M NFA significantly decreased as compared to the untreated group (**p < 0.01) (Figure 3A). A significant decrease (**p < 0.01) was also observed in the mean VSL of sperm moving toward the ascending gradient of NFA, after treatment with the antagonist (Figure 3B). A frequency distribution plot for the VSL of the sperm population exposed to the antagonist showed a significant change in sperm VSL. In the absence of pre-treatment with ICI-118,551, a higher percentage of sperm responded to NFA with VSL 81-120 and >120 μm/sec compared to those seen with DMEM alone. However, the percentage of sperm responding to NFA with VSL 81-120 and >120 μm/sec was significantly lower in sperm pre-treated with the antagonist (Figure 3C). Data for each experimental set are provided in Supplementary Table S2.

### NFA increases [Ca^2+^]_I_ levels

**FIGURE 4:**
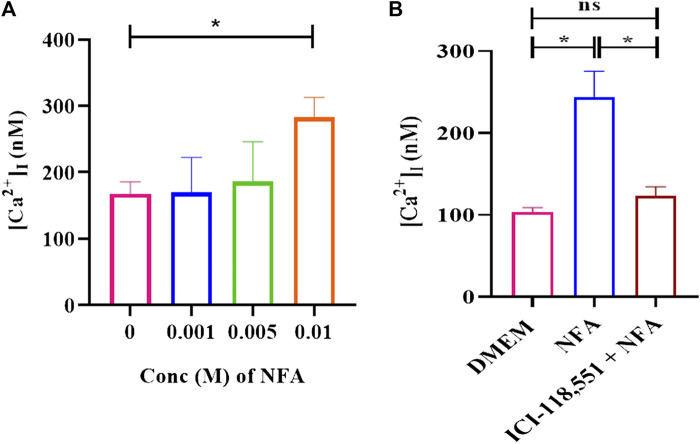
NFA increases [Ca^2+^]_I_ levels in capacitating sperm. [Ca^2+^]_I_ was assessed on exposure of capacitating sperm to 0.001, 0.005, and 0.01 M NFA using Fura-2 AM-based assay. Fura-2AM-loaded capacitating sperm were stimulated with the stated concentrations of NFA and the corresponding F-max and F-min values were recorded after the addition of 1% Triton X-100. Excitation of the dye with bound and unbound calcium was measured at 340 and 380 nm, respectively, and fluorescence emission at 500 nm. The figure shows a graph of [Ca^2+^]_I_ at different concentrations of NFA **(A)**. The effect of the β-2-AR antagonist on [Ca^2+^]_I_ was studied after 30 min exposure of capacitating sperm to 0.01 μM ICI-118,551 before exposing them to 0.01 M NFA **(B)**. The results presented are cumulative from three experiments. Values are expressed as mean ± SEM. *p < 0.05.

To determine the influence of 0.01 M NFA on [Ca^2+^]_I_, Fura-2AM-loaded capacitating sperm were incubated with 0.001, 0.005, and 0.01 M NFA and the fluorescence intensity was measured to calculate the levels of [Ca^2+^]_I_. A dose-associated increase in [Ca^2+^]_I_ was observed in sperm exposed to 0.001, 0.005, and 0.01 M NFA, with the increase being significant at 0.01 M NFA compared to DMEM (*p < 0.05) (Figure 4A). This increase was abrogated when capacitating sperm treated with ICI-118,551 for 30 min were exposed to 0.01 M NFA. They were comparable to the levels in capacitating sperm (Figure 4B). Data of each experimental set are provided in Supplementary Tables S3A,B.

### NFA does not induce capacitation or acrosome reaction in sperm

**FIGURE 5:**
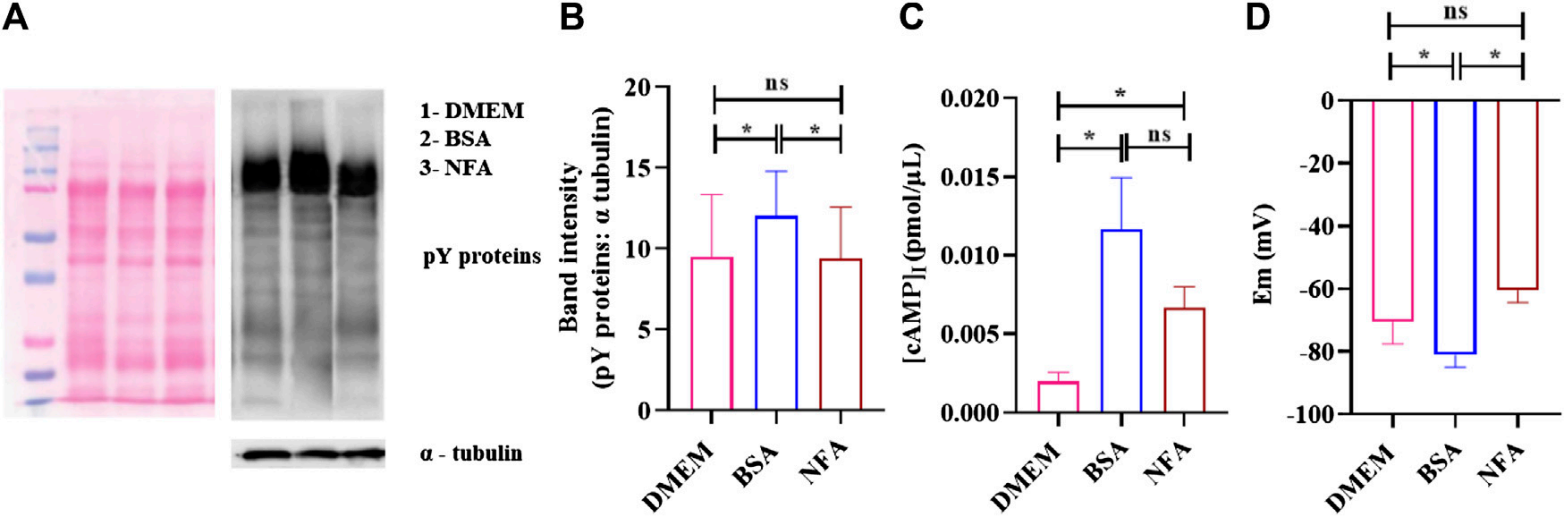
NFA at chemotactic concentrations does not induce capacitation. Protein tyrosine phosphorylation, [cAMP]_I_, and membrane potential was determined in sperm incubated in DMEM, 2.5% BSA, or 0.01 M NFA for 2.5 h. Protein tyrosine phosphorylation was detected by Western blot analysis using mouse anti-phosphotyrosine and HRP-conjugated goat anti-mouse as primary and secondary antibodies, respectively. A representative image of the Western blot for pY proteins (right) and its corresponding ponceau-stained blot (left) are shown **(A)**. The band intensities of tyrosine-phosphorylated proteins normalized to that of the loading control, α- tubulin, are shown **(B).** [cAMP]_I_ was assessed by competitive ELISA. Absorbance was measured at 480 nm. The figure shows a graph of [cAMP]_I_ levels under different conditions **(C)**. The sperm membrane potential was determined using DiSC3-based fluorimetric assays. Represented are absolute Em (mV) values of the sperm membrane after treatment **(D)**. Values represent the mean ± SD of three independent experiments. (*p < 0.05; **p < 0.01).

Sperm capacitation and acrosome reaction are prerequisites for successful fertilization. We explored the effect of 0.01 M NFA on these parameters. The effect on capacitation was evaluated by determining the extent of tyrosine phosphorylation, membrane potential, and levels of intracellular cAMP in sperm after exposure to NFA. For all these parameters, 2×10^6^ sperm were incubated for 2.5 h in DMEM alone, or DMEM supplemented with either 2.5% BSA or 0.01 M NFA. Incubations with BSA served as a positive control for capacitation. The extent of tyrosine phosphorylation was detected using a specific anti-phosphotyrosine antibody by Western blot analysis (Figure 5A) and IIF. Sperm did not show any significant difference in the phosphotyrosine intensity after 2.5 h of incubation with NFA (Figure 5B). Interestingly, detection by IIF showed a slight yet significant decrease in the percent of sperm with tail fluorescence (Supplementary Figure S2).

[cAMP]_I_ was measured using competitive ELISA. The [cAMP]_I_ increased after treatment with NFA (*p < 0.05), reaching a concentration of 0.006 pmol/μL as against 0.002 pmol/μL seen on incubation with DMEM (Figure 5C). Hyperpolarization of the sperm plasma membrane was also studied using a fluorimetric population assay to determine the absolute membrane potential (Em). Em was determined using a positively charged carbocyanine probe DiSC_3_. Sperm incubated with BSA displayed Em of -81.17 ± 3.09 mV as against -70.55 ± 5.68 mV seen in DMEM incubation. NFA was least effective in inducing capacitation-associated hyperpolarization with an Em of -60.47 ± 3.20 mV. The Em of sperm treated with 0.01 M NFA was found to be comparable to that seen with DMEM (Figure 5D).

**FIGURE 6:**
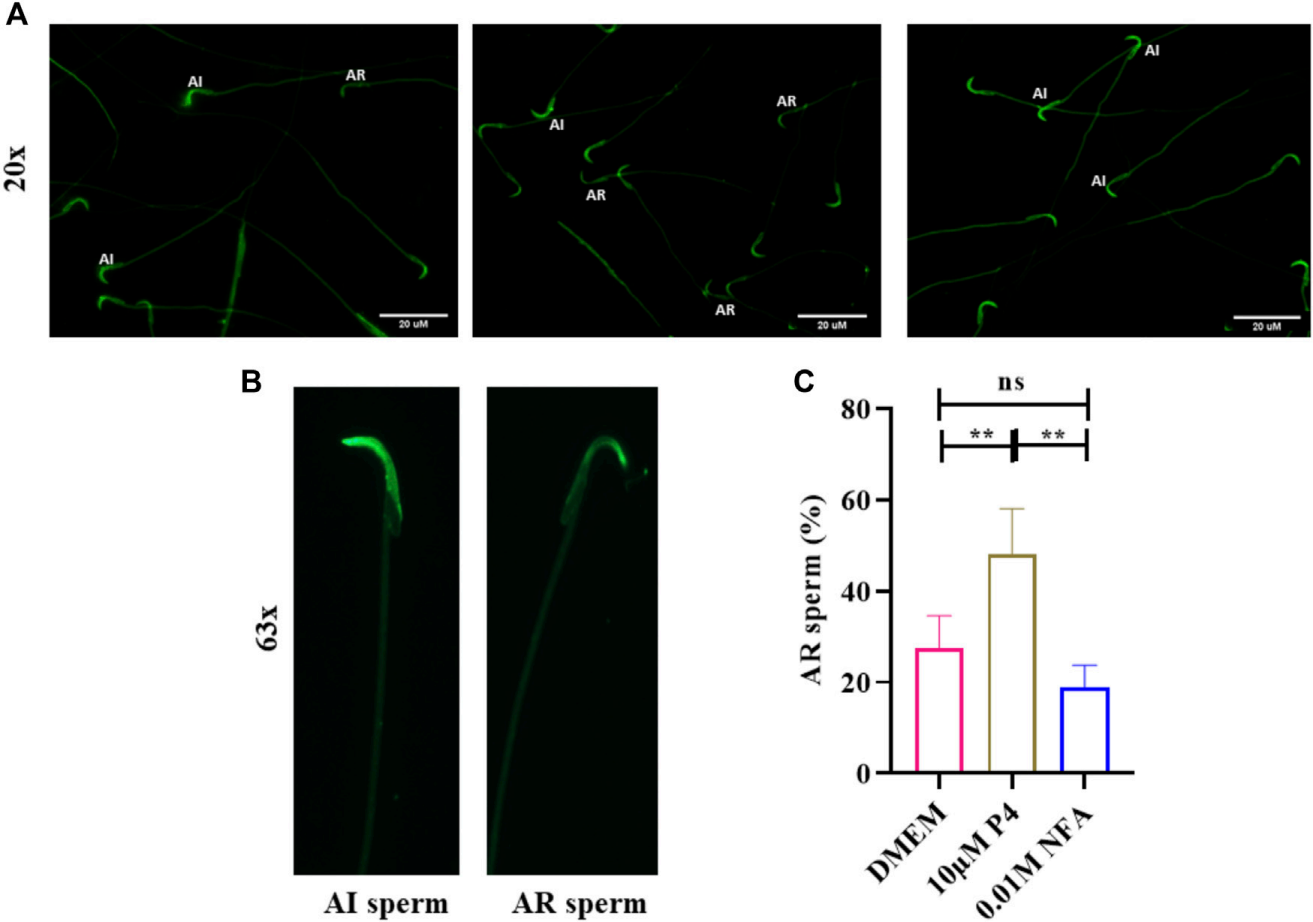
NFA does not induce acrosome reactions in sperm. The acrosome reaction was evaluated by PSA-FITC staining. Sperm were incubated in DMEM, without or with 10 μM progesterone (P4), or 0.01 M NFA for 1 h after capacitation and subjected to PSA-FITC staining. After PSA-FITC staining, sperm were examined by epifluorescence microscopy. AR, acrosome reacted sperm; AI, acrosome intact sperm **(A,B)**. The percentages of acrosome reacted sperm were calculated by dividing the number of acrosome reacted sperm by the total number of sperm for each group **(C)**. Bars represent mean ± SD.

To evaluate whether NFA induces an acrosome reaction, 2×10^6^ capacitating sperm were incubated with 0.01 M NFA for 1 h and then assessed by PSA-FITC staining. Sperm incubated with 10 μM progesterone were used as a positive control. At the end of the incubation period, the motility was evaluated and was observed to be greater than 70% in each group. PSA-FITC staining uses fluorescein-conjugated lectin to distinguish sperm with an intact acrosome (AI) from those with a reacted acrosome (AR). Sperm with intact acrosomes display uniform and bright fluorescence whereas acrosome reacted sperm show less fluorescence in the sperm head. The percentage of AR sperm in the NFA-treated suspension was comparable to that seen with DMEM. Incubations with 10 μM progesterone showed a significantly higher percentage of AR sperm than expected (Figure 6).

## Discussion

In mammals, sperm chemotaxis is an important guidance mechanism directing sperm to the egg. Human sperm (Gnessi et al., 1986a), neutrophils (Naccache et al., 2000a; Southgate et al., 2008a), and bacterial species such as *Pseudomonas aeruginosa* (Ijiri et al., 1994a; Bufe et al., 2015a) and *E. coli* (Bi et al., 2013a) have all been reported to respond chemotactically to synthetically derived N-formylated peptides. Recent studies by our group have reported significantly higher levels of NFA in the ovulatory phase-oviductal fluid compared than in the pre-ovulatory phase. Additionally, we demonstrated it to be a sperm chemoattractant at 0.01 M concentration (Bhagwat et al., 2021a). In the present study, we investigated the response of sperm to gradients ranging from 0.001–0.02 M NFA using microfluidics-based chemotaxis assays. Whilst the percentage of sperm moving toward higher concentrations and their velocities increased concomitant to the gradient concentrations, it was highly significant at a gradient concentration of 0.01 M NFA. A significant increase in the percentage of sperm with a VSL range of 81–160 and >160 μm/sec was noted with 0.01 M NFA. At 0.02 M NFA, there was a drop (Figures 1A–C). This bell-shaped dose-response curve is characteristic of a chemoattractant. These observations indicate that sperm can sense increasing concentration gradients up to 0.01 M NFA; any further increase is detrimental, thereby suggesting the possibility that NFA is sensed by the sperm via some receptor and that higher concentrations of NFA probably saturate and desensitize the receptors, rendering the sperm unable to sense it resulting in a drop in velocities and, consequently, the number of chemotactic sperm.

We, therefore, investigated the cognate receptors for NFA on sperm. NFA has been patented as a β-2-AR agonist (US-2005261338-A1, US-2007179179-A1, and US-2008269344-A1). β-2-ARs are transmembrane glycoproteins containing eight alpha helices (3-extracellular and 5-intracellular) and are associated with a heterotrimeric G-protein (specifically a G-protein stimulatory subunit) (Yang et al., 2021). β-2-ARs have been reported in mouse and human sperm and have a molecular weight of approximately 72 kDa (Adeoya-Osiguwa et al., 2006). We looked for its presence in rat sperm and, using β-2-AR-specific antibody, detected a distinct band at approximately 72 kDa (Figure 2A). The expression of β-2-AR did not differ between capacitating and non-capacitating sperm. Localization by IIF showed that β-2-ARs are localized on the head and in the mid-piece region of the sperm, with decreasing or fainter fluorescence along the principal piece and end piece. Previously, β-2-ARs have been reported to be localized around the acrosomal cap, head, and neck, along with less intense localization around the tail (Adeoya-Osiguwa et al., 2006). Loss of the positive signal on permeabilization of the sperm membrane indicated its presence on the sperm surface (Figure 2B). This observation suggested that β-2-AR may be the putative receptor for NFA.

To further confirm this possibility, sperm chemotaxis to NFA was determined using ICI-118,551, a β-2-AR specific antagonist. ICI-118,551 is reported to be effective at a concentration of 10 nmol/L in somatic cells (Pecha et al., 2015). We first determined the lowest concentration of the antagonist that was non-toxic to sperm, by checking sperm viability and motility at different concentrations of ICI-118,551 and at time intervals of 15 min from 0–45 min. The effect of ICI-118,551 on sperm motility and viability was found to be in the order 1 µM > 0.1 µM > 0.01 µM with 0.01 M ICI-118,551 showing no detrimental/least detrimental effect on sperm viability (Tables 1, 2). Hence, the chemotactic effect of NFA was tested on sperm pre-treated with 0.01 µM ICI-118,551 for 30 min. A significantly reduced chemotactic response of sperm to NFA affirmed that NFA mediates chemotaxis via the β-2-AR receptors present on sperm (Figure 3).

Ca^2+^ is one of the important ions involved in different sperm processes such as capacitation and acrosome response (Correia et al., 2015). [Ca^2+^]_I_ levels have been found to increase during sperm chemotaxis (Teves et al., 2009; Bhagwat et al., 2018). We observed an increase in [Ca^2+^]_I_ in capacitating sperm after treatment with 0.01 M NFA. This increase was abrogated in the presence of ICI-118551 (Figures 4A,B). Intriguingly, we observed a significant decrease in [cAMP]_I_, when capacitating sperm were exposed to 0.01 M NFA for 30 min (**p < 0.01; Supplementary Figure S3). However, the intracellular levels of Ca^2+^ were elevated (Figure 4). As per the canonical pathway, β-2-AR signaling occurs through the G_S_-adenylyl cyclase–cAMP pathway wherein cAMP increases. It is possible that NFA acts through β-2-AR via the non-canonical pathway. This has been reportedly seen in HEK-293 cells wherein β-2AR-mediated Ca^2+^ influx has been shown to occur independently of conventional cAMP-dependent signaling (Galaz-Montoya et al., 2017). The elevation of [Ca^2+^]_I_ observed by us on exposure of capacitating sperm to NFA may regulate flagellar curvature toward symmetrical beating reported to occur in chemotaxis as opposed to asymmetrical beating that occurs during hyperactivation (Chang and Suarez, 2010).

As capacitation, chemotaxis, and acrosome reaction are tightly associated events, we also investigated whether or not NFA may have a role in these important events. Toward this, we analyzed tyrosine phosphorylation, [cAMP]_i_ and absolute Em of sperm after treatment with NFA as these parameters are known to be affected during capacitation (Puga Molina et al., 2018). Studies have shown the increased association of tyrosine phosphorylation with hyperactivated motility (Nassar, 1999; Y.-Y. Wang et al., 2021). Our results showed that NFA *per se* did not affect the levels of protein tyrosine phosphorylation (Figures 5A,B). However, with NFA, a slight but significant increase in the [cAMP]_i_ levels compared to DMEM was noted (Figure 5C). This increase in cAMP is not surprising as on incubation of uncapacitated sperm with NFA for 2.5 h, there is a slight but significant increase in motility compared to that seen in DMEM at 0 h (data not shown). Tyrosine phosphorylation is increased only on capacitation. Hence, we see it with BSA which was used as a positive control of capacitation, but not with NFA in which case it is as much as seen with DMEM, thus providing evidence that NFA does not induce capacitation. Membrane hyperpolarization is also a hallmark of capacitation (Baro Graf et al., 2019). Thus, it can be seen in incubations with BSA (Figure 5D). A slight yet significant depolarization is seen with NFA in comparison to that seen with DMEM, thus affirming that NFA does not induce capacitation. However, the possibility that NFA may inhibit capacitation cannot be ruled out. An acrosome reaction is a prerequisite for successful fertilization. It is well-known that spermatozoa that undergo premature acrosome reactions are unable to recognize a chemoattractant gradient (Guidobaldi et al., 2017) and are unable to fertilize the egg (Barbonetti et al., 2008). The acrosome was not affected by 0.01 M NFA (Figure 6). Taken together, all these observations suggest that 0.01 M NFA may not be involved in capacitation or acrosome reactions.

**FIGURE 7:**
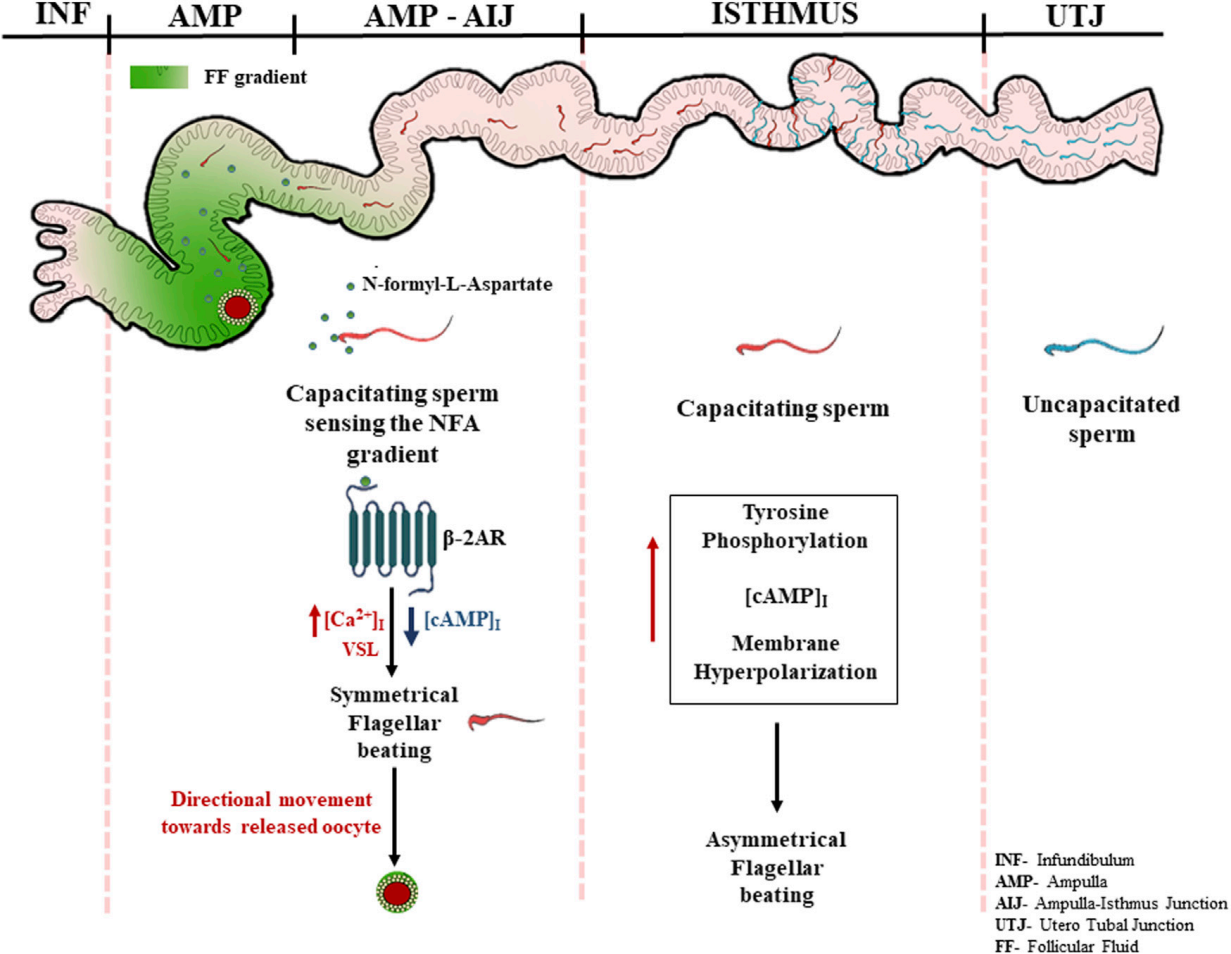
Schematic representation for the mechanism of chemotaxis mediated by NFA.

Based on our knowledge of sperm signaling events from the literature and our own observations, we propose a putative mechanism of chemotaxis mediated by NFA. The high levels of progesterone during ovulation may induce capacitation of the sperm parked within the oviduct, thereby inducing their hyperactivation and release from the sticky milieu of the oviduct following which NFA released into the oviduct during ovulation may direct them toward the oocyte via its interaction with β-2-AR present on the sperm surface, causing an additional increase in the intracellular levels of Ca^+2^. Increased levels of Ca^+2^ may result in Ca^+2^-associated symmetrical flagellar beating causing the sperm to move in a linear trajectory with increased velocity toward the egg (Figure 7).

In summary, we report that NFA triggers chemotaxis in sperm and does not induce capacitation or acrosome reactions. NFA mediates its chemotactic effect *via* β-2-AR present on the sperm surface possibly through non-canonical signaling, thereby increasing sperm intracellular calcium levels over and above those seen on capacitation and influencing the linear swimming of the sperm toward the oocyte. This study, thus, ignites the possibility of using NFA as a chemoattractant to select good quality sperm for enhancing the “take home baby” rate of IVF procedures.

## Data Availability

The original contributions presented in the study are included in the article/Supplementary Material; further inquiries can be directed to the corresponding author.
