# Supplementary figures and images for: N-Formyl-L-aspartate mediates chemotaxis in sperm via the beta-2-adrenergic receptor
Source: Front Cell Dev Biol. 2022 Sep 23;10:959094. doi: 10.3389/fcell.2022.959094 (PMC9538769; doi:10.3389/fcell.2022.959094)

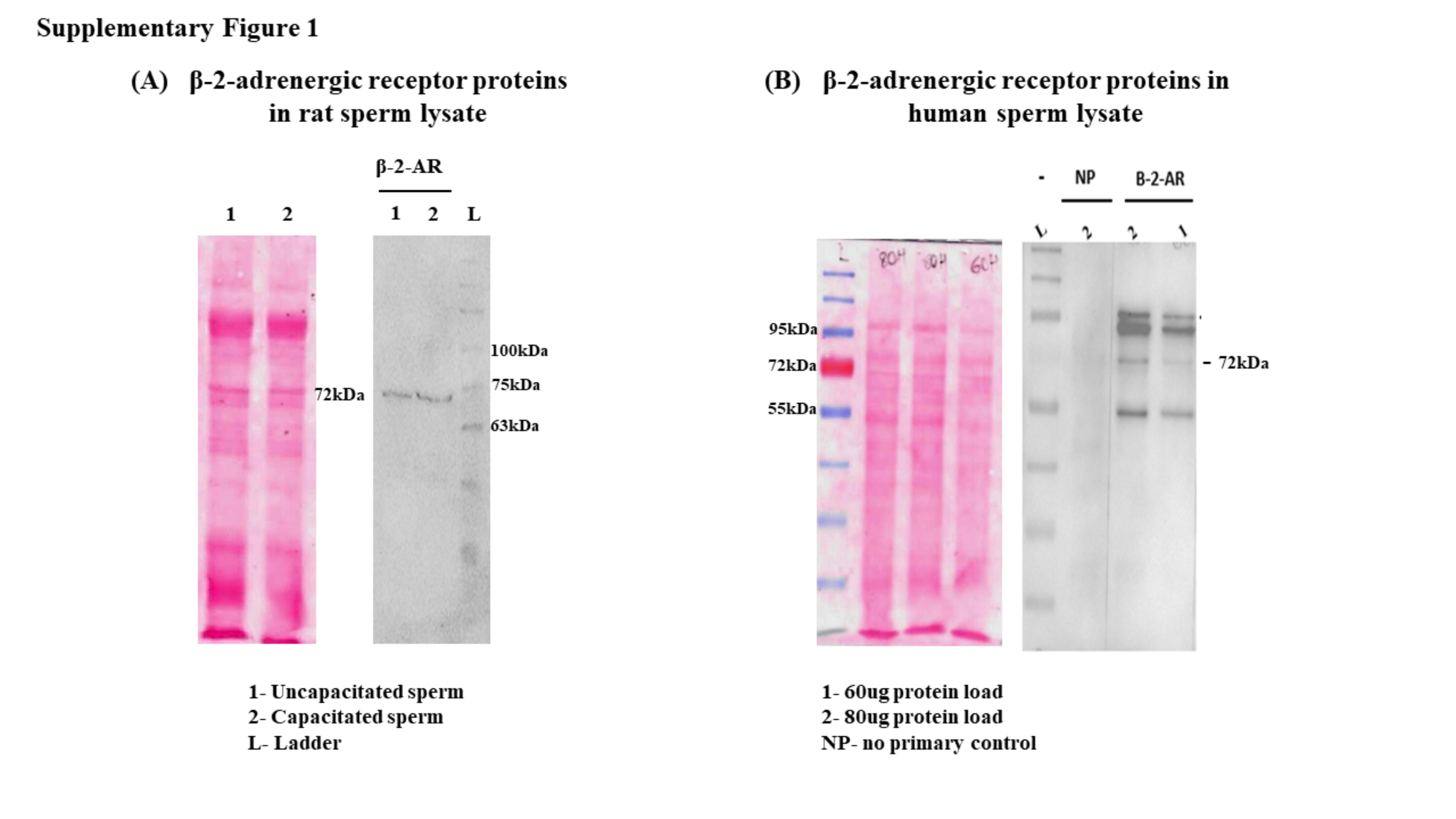

Supplement: Supplementary file 2 [file Image1.TIFF]

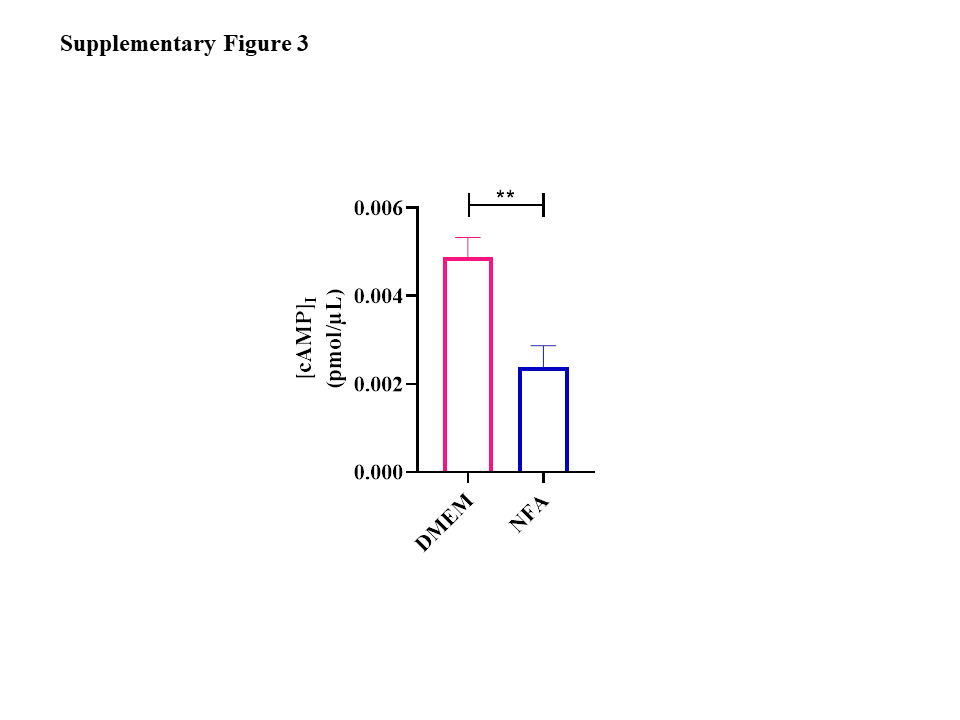

Supplement: Supplementary file 4 [file Image3.TIF]

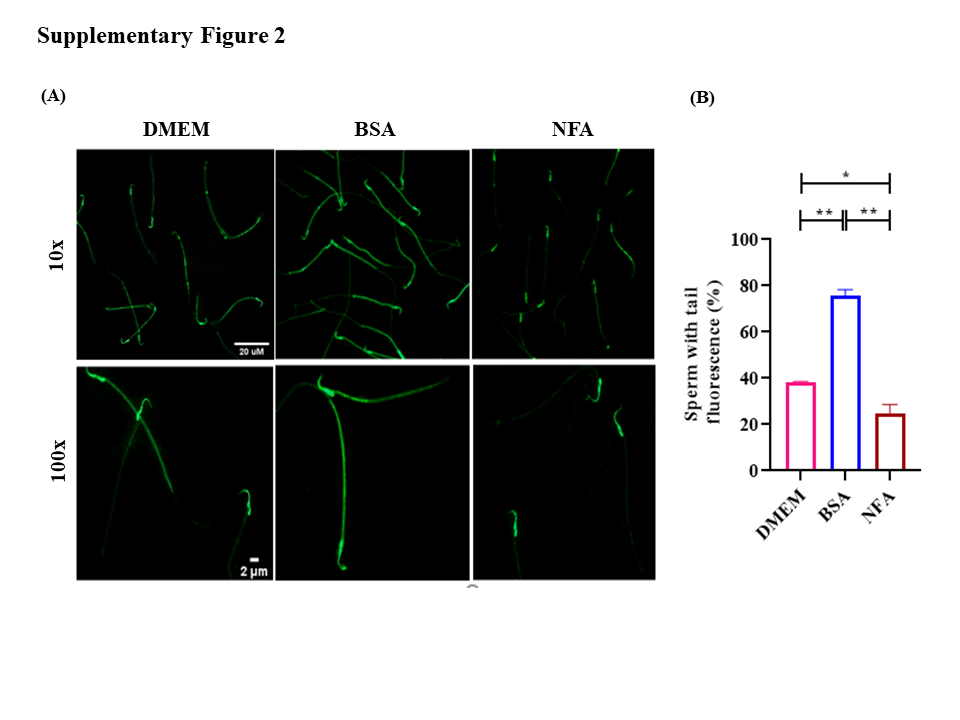

Supplement: Supplementary file 5 [file Image2.TIF]
